# The Importance of Genetic Testing: A Case Report of Wilson's Disease in Two Siblings of a Three-Sibling Family

**DOI:** 10.7759/cureus.77891

**Published:** 2025-01-23

**Authors:** Siva Govindan, Jennie Santhanam, Meenakshi Sundari S N, Jeyapriya U, Bolisetty Shanmukha Sai

**Affiliations:** 1 General Medicine, Sri Ramaswamy Memorial Medical College Hospital and Research Centre, Kattankulathur, IND

**Keywords:** atp7b mutation, copper, genetic testing, wilson disease, wing-beating tremor

## Abstract

Mutations in the adenosine triphosphatase (ATPase) copper transporting beta (ATP7B) gene result in Wilson's disease (WD), a rare autosomal recessive condition that affects copper metabolism, leading to its accumulation in multiple tissues, including the liver and the brain. This case report details the clinical presentation of three siblings born out of a consanguineous marriage, each displaying different manifestations. The youngest sibling exhibited significant hepatic and neurological symptoms, the middle sibling experienced neuropsychiatric issues, and the eldest one initially showed psychological distress without classic symptoms of WD. Genetic testing confirmed WD in the symptomatic siblings and ruled it out in the eldest, guiding their personalized treatment plans and reducing psychological stress. This case emphasizes the critical role of genetic testing in the early diagnosis, management, and familial risk assessment of WD. Additionally, it highlights the necessity of a comprehensive approach that includes medical, psychological, and social support to enhance the prognosis of the illness.

## Introduction

Wilson's disease (WD) is a hereditary condition that impairs hepatic copper transport and causes subsequent deposits of copper in multiple organs [1]. It represents a significant health burden worldwide, with a prevalence ranging from 1 in 30,000 to 1 in 1,00,000 individuals [2]. Broader genetic screening yielded an estimated carrier frequency of 1 in 5,000, indicating that the true prevalence may be greater. While WD typically presents with hepatic and neurological symptoms, its clinical spectrum can vary widely. A favourable family history, which is frequently connected to a high frequency of consanguineous marriages, is linked to over half of WD cases in India [3]. Additionally, limited access to healthcare services and diagnostic facilities in certain areas may contribute to the under-reporting and under-diagnosis of WD cases. Genetic testing has emerged as a valuable tool in confirming WD diagnosis, guiding treatment decisions, and assessing familial risk. A predisposition for the female sex is frequently observed in paediatric WD. A predisposition for the female sex is commonly observed in paediatric Wilson illness [4]. The typical features of hepatic Wilson were deep jaundice, age less than 22, female predilection, tremors, absence of hepatic encephalopathy, haemolysis, and low platelets. Low serum ceruloplasmin levels are a hallmark of WD [5]. Neurological Wilson is characterised by movement disorders like tremor, dystonia, parkinsonism, and ataxia, along with symptoms such as drooling, irritability, and occasional seizures. Here we describe a comprehensive case report of WD in a family of three siblings with diverse clinical manifestations. The pivotal role of genetic testing in their management is reinforced in our report.

## Case presentation

**Figure 1:**
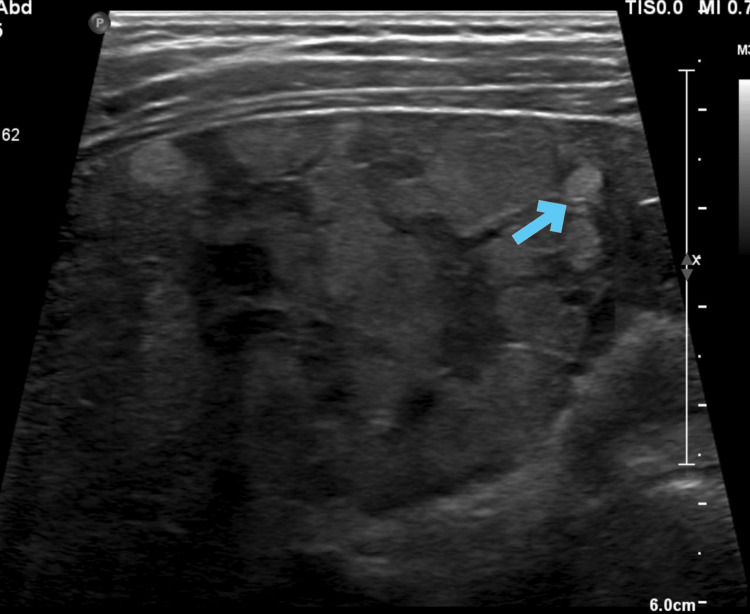
Ultrasonography of the abdomen of the youngest sibling The image shows features of chronic liver disease with regenerating nodules (blue arrow)

**Figure 2:**
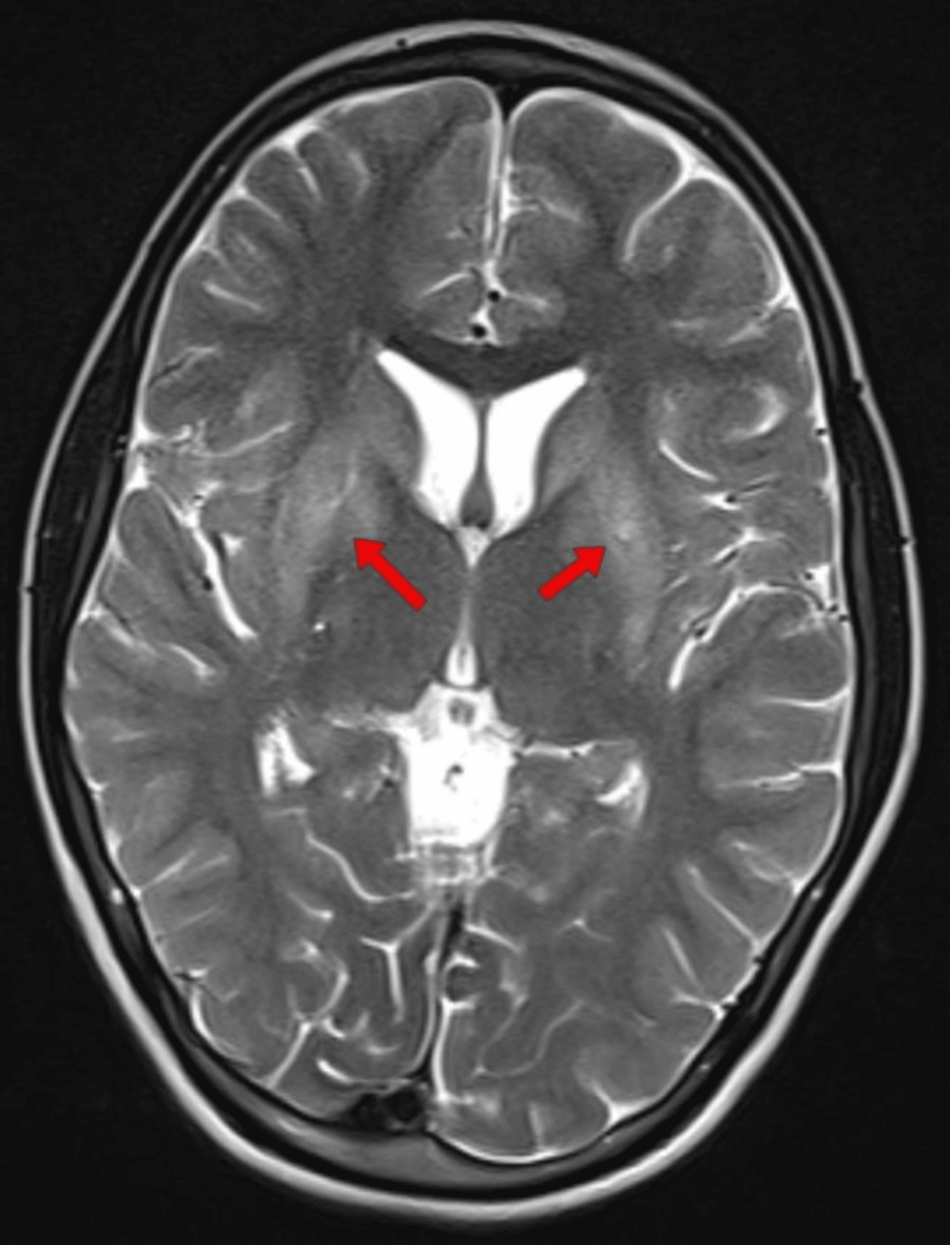
MRI brain of the youngest sibling MRI, Magnetic Resonance Imaging; T2-weighted image of the brain showing bilateral basal ganglia hyperintensity (red arrows).

Three siblings, aged 14, 17, and 21, born out of second-degree consanguinity, presented to us with varying clinical features of WD. The 14-year-old female patient, the youngest of the three siblings, presented with yellowish discoloration of the eyes and abdominal distension. She also had a history of unsteadiness while walking. An examination of the abdomen showed hepatosplenomegaly with shifting dullness. A neurological examination revealed a classical wing-beating tremor. She also had spells of incessant crying with generalized irritability and drooling. Laboratory investigations revealed an altered liver function test with the aspartate transaminase (AST) at 139 U/L, alanine transaminase (ALT) at 111 U/L (normal range for both=7-40 U/L), increased total bilirubin level of 2.5 mg/dL (normal range=0.3-1.2 mg/dL) with a direct bilirubin level of 1.3 mg/dL (n0.0-0.3 mg/dL). She had elevated urine copper excretion at 1426 µg/day (normal range=20-50 µg/day), low ceruloplasmin at 1.9 mg/dL (normal range=20-60 mg/dL), and a high serum copper level at 2.86 mg/dL (normal range=0.9-1.9 mg/dL). Kayser-Fleischer (KF) rings were found on ophthalmologic examination, consistent with WD. Treatment with zinc, D-penicillamine (DPA), and antimuscarinic agents led to symptomatic improvement. The associated radiological images are shown in Figures 1, 2.

**Figure 3:**
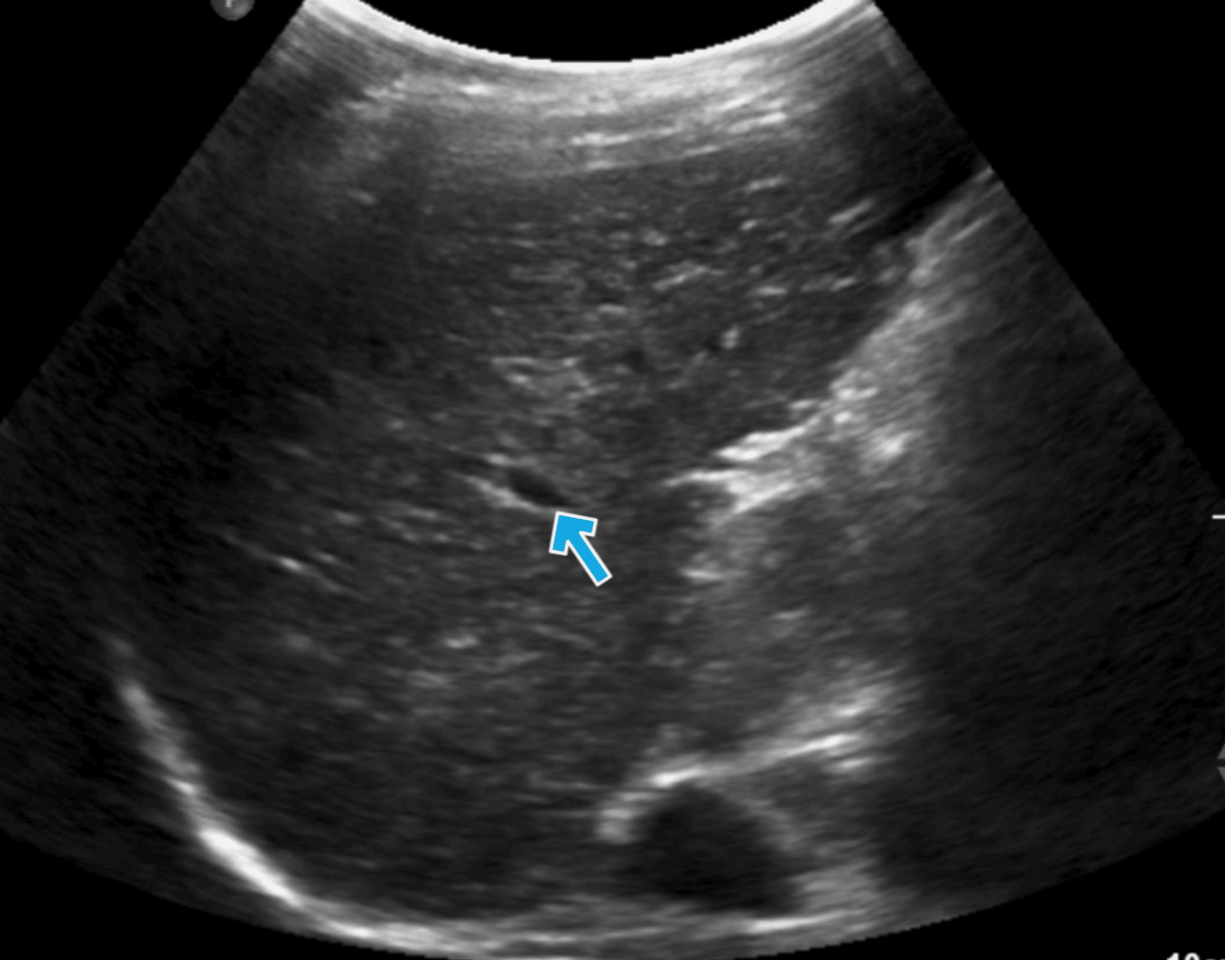
Ultrasonography of the abdomen of the middle sibling The image shows chronic liver disease with altered liver echoes. The arrow indicates the altered echo texture with regenerating nodules.

**Figure 4:**
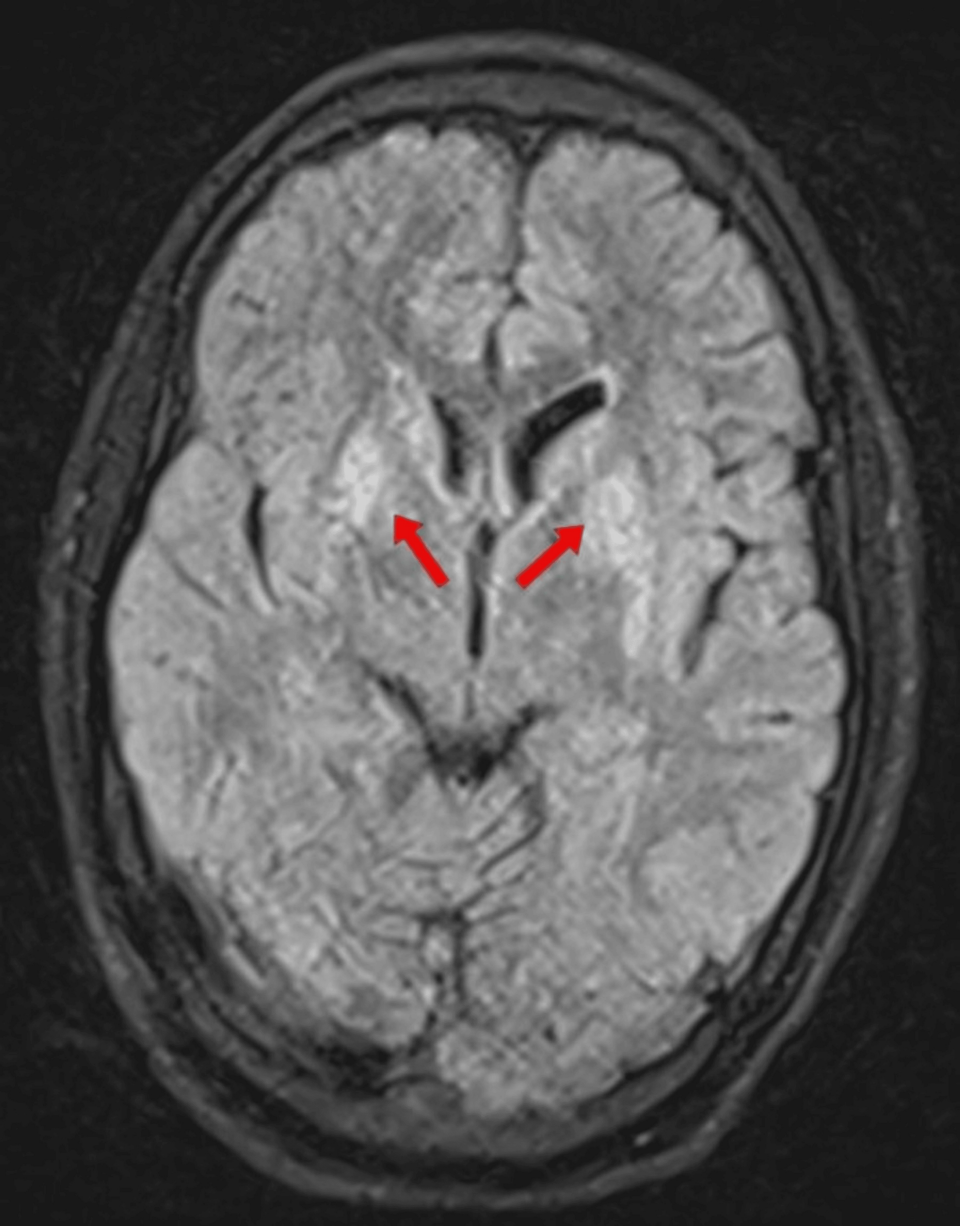
MRI brain of the middle sibling MRI, Magnetic Resonance Imaging; FLAIR, Fluid attenuated inversion recovery; T2-FLAIR showing bilateral basal ganglia hyperintensity.

The second sister, aged 17, was brought to us with complaints of increased anxiety and fear of social gatherings. Her parents also gave a history of increasing complaints from school citing poor scholastic performance, restlessness during classes and difficulty concentrating. On physical examination, no yellowish discoloration of the eyes was noted. However, an ophthalmological examination revealed a KF ring. She also had tremors. Her liver functions tests were normal. Her ceruloplasmin level was low at 2.3 mg/dL (normal range=20-60 mg/dL), urinary excretion of copper was elevated at 1274 µg/day (normal range=20-50 µg/day), and blood copper level was high at 2.43 mg/dL (normal range=0.9-1.9 mg/dL). Treatment initiation with zinc supplementation and DPA resulted in symptom stabilization. The results of her radiological imaging tests are shown in Figures 3, 4.

Furthermore, genetic sequencing of the protein coding region in genes associated with inherited disease was performed in peripheral venous blood using next generation sequencing (NGS) with Illumina NovaSeq 6000 sequencer (Illumina, Inc., San Diego, CA, USA) showed that both the younger sisters were homozygous for a variant of uncertain significance (VUS) in ATP7B c.3045G>A (p.Leu1015=). A VUS in the ATP7B gene does not provide a definitive diagnosis for WD, as its impact on the condition is uncertain, and genetic testing may not capture factors such as copy number variants (CNV), intronic variants, or other possible mutations. However, due to the strong clinical evidence, the two younger siblings were diagnosed with WD.

The eldest sister, aged 21, did not have any clinical symptoms or signs of WD and was not evaluated. Six months later, she presented to us with depression and suicidal ideation with deliberate self-inflicted cut injury on her wrist. Though the features were identical to the neuropsychiatric manifestation of WD, her serum ceruloplasmin levels were found to be within normal limits. No significant ophthalmological findings were present. Upon detailed psychological evaluation, she was diagnosed with clinical depressive disorder triggered by the fear of suffering from WD like her siblings. She was started on antidepressants. We decided to subject her to genetic testing to alleviate her fears. The test revealed no significant variations in the ATP7B gene. There was no other significant family history. The parents were advised to undergo genetic counseling but were unwilling for the same.

## Discussion

WD is an uncommon autosomal recessive disorder resulting from mutations in the ATP7B gene, responsible for encoding a copper-transporting adenosine triphosphatase (ATPase). This genetic anomaly disrupts hepatic copper transport, leading to toxic copper deposition primarily in the brain and the liver [6]. Despite being uncommon, it has a significant impact on the affected patients and their families. Although it can appear at any age, the condition first becomes apparent in the teenage years or early adulthood. The age of onset has also been linked to polymorphisms in the methylenetetrahydrofolate reductase (MTHFR) gene; however, other research studies do not corroborate this [7]. Classically, paediatric-onset WD is associated with hepatic manifestations, and adult-onset WD is linked to neurological manifestations [8]. Rkain et al. studied 24 paediatric WD patients in eastern Morocco. They identified three major obstacles to detecting WD: a delay in seeking consultation, clinical heterogeneity, and the fast-changing course of the disease [9]. 

Physiologically, the ATP7B gene aids in the transport of copper into the trans-Golgi Network (TGN) which is further incorporated into ceruloplasmin or excreted into bile. In WD, due to the absence of functional ATP7B, the transport of copper into TGN is affected. This leads to the accumulation of cytosolic copper, which is taken up by the lysosomes. When the lysosomes are saturated, they burst and release the copper into the cytosol. This leads to free radical damage and causes an apoptotic chain reaction in the cell that releases free copper into the blood [10]. 

Hepatic manifestations of WD range from incidental findings of abnormal liver function tests to severe conditions like acute liver failure (ALF). In the early stages, patients might not have any symptoms but show deranged liver enzymes or abnormal ultrasonography findings. As the condition progresses, manifestations of chronic liver disease, such as hepatomegaly, splenomegaly, and cirrhosis leading to ascites and variceal bleeds, may occur. In our case, the youngest sibling presented with deranged liver function tests and hepatosplenomegaly, indicating significant hepatic involvement, pointing towards a WD diagnosis. ALF-like presentation in WD is characterized by hepatomegaly, jaundice, and coagulopathy, with or without encephalopathy in previously-healthy children. The Paediatric Acute Liver Failure (PALF) study group defines ALF as an international normalized ratio (INR) of ≥1.5 with encephalopathy or an INR of ≥2.0 without encephalopathy. A few children may have a history of acute self-limited hepatitis-like illness with recurrent jaundice, haemolytic anaemia, or elevated transaminases. This presentation is often considered ‘acute-on-chronic liver failure’, as liver histology typically shows pre-existing chronic damage. The youngest sibling’s presentation, with abdominal distension, jaundice, and altered liver enzymes, along with the radiological findings fits the profile of ‘acute-on-chronic liver failure’ seen in WD [11].

**Figure 5:**
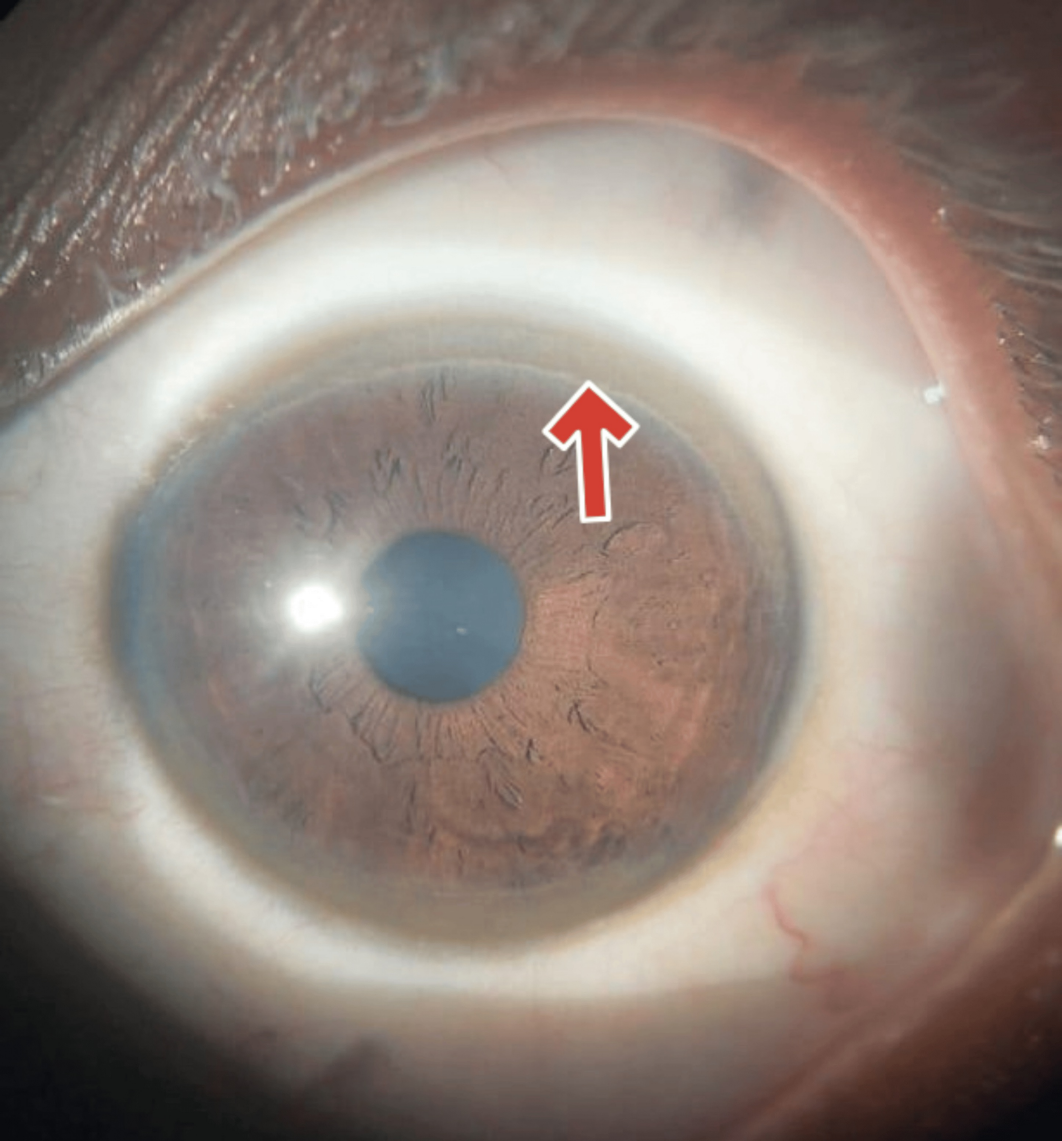
Clinical image of the Kayser-Fleischer (KF) ring in the youngest sibling

**Figure 6:**
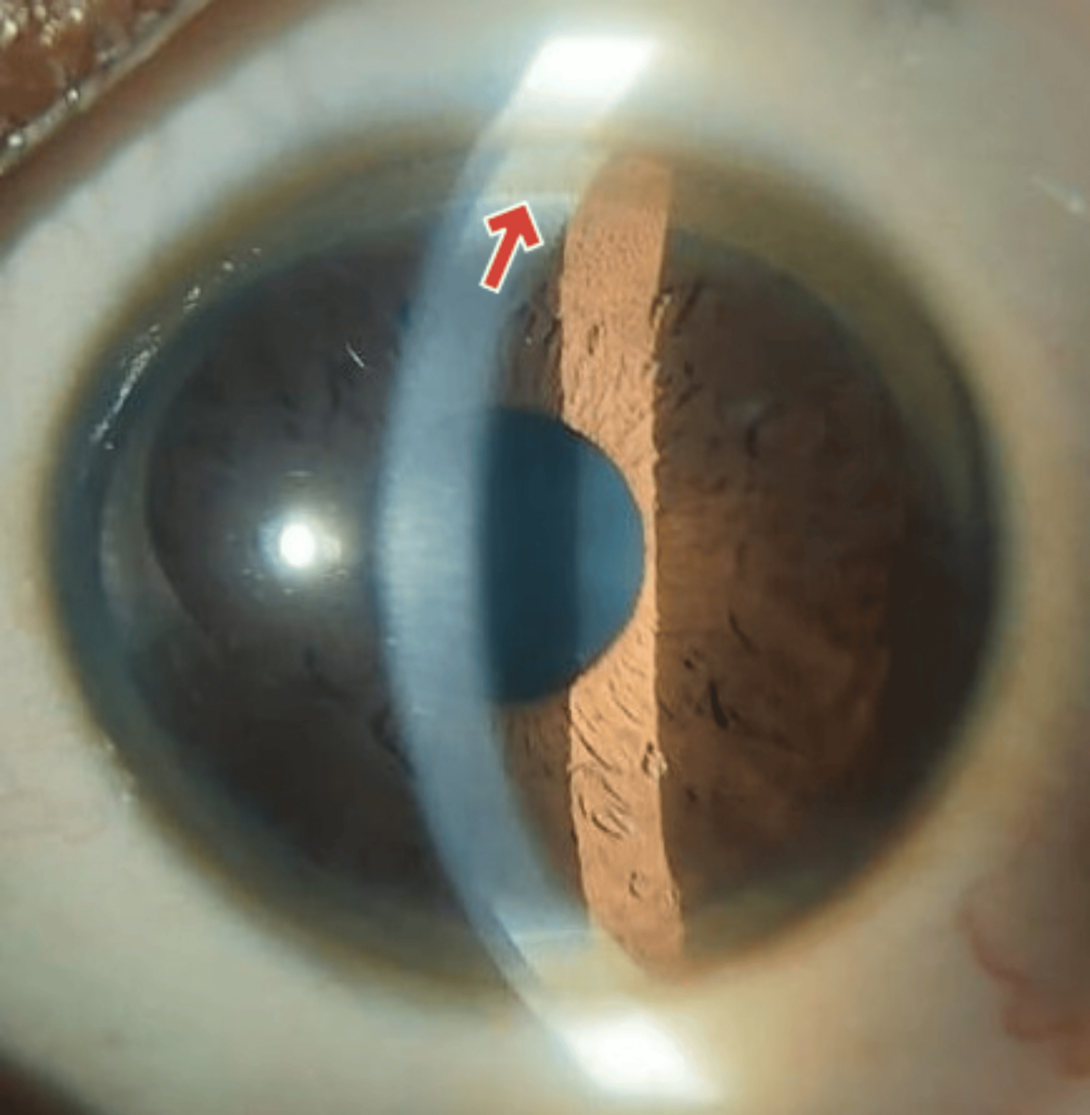
Clinical image of the Kayser-Fleischer (KF) ring seen in the middle sibling

The accumulation of copper in the Descemet's membrane causes the KF ring, which appears as green, brown, or golden pigmentation encircling the cornea. KF rings are seen in all individuals with neurological WD, 40-50% of patients with hepatic WD, and 20-30% of pre-symptomatic WD cases. In the cases under discussion, the youngest and the middle siblings were found to have KF rings, confirming the presence of WD despite differing clinical presentations (Figures 5, 6).

Neurological manifestations are the most common clinical presentations of WD and they usually include a spectrum of movement disorders, with tremor seen in almost half the patients at diagnosis. Tremors could be postural, resting, or kinetic and often involve the upper extremities but can spread to the legs, head, or the entire body. The youngest sibling exhibited neurological symptoms, including unsteadiness while walking and a wing-beating tremor. Dystonia can be the initial neurological manifestation in 11-65% of patients. A characteristic dystonic feature is ‘Risus sardonicus,’ an abnormal, fixed smile due to dystonia of the facial muscles. Parkinsonism, reported in 19-62% of patients, includes bradykinesia, dysarthria, difficulty in swallowing, rigidity, gait abnormality, and drooling. Ataxia, involving cerebellar dysfunction, is found in about 30% of patients. In addition to the wing-beating tremor, the youngest sibling also experienced drooling and spells of incessant crying with generalized irritability, which align with the neurological and psychiatric symptoms of WD. Drooling, observed in nearly 68% of neurological cases, is often due to dysphagia or orofacial dystonia. Epilepsy seen in 6.2-8.3% of WD patients, with the commonest type of seizure being generalized seizure. Early psychiatric signs of WD can even appear in childhood, manifesting as decreased academic performance, inappropriate behaviour, or impulsiveness. Mood disorders are the commonly-observed psychiatric manifestation of WD [12].The middle sister in our case exhibited increased anxiety and fear of social gatherings, along with poor scholastic performance and difficulty concentrating. These are consistent with early psychiatric signs of WD. The clinical manifestations of WD are listed below (Table 1). 

**Table 1:** Clinical manifestations of Wilson disease

Organ system	Manifestations
Liver [5]	Splenomegaly (haemolysis or portal hypertension), steatosis, acute hepatic failure, chronic hepatitis, liver failure with portal hypertension
Neurological [5]	Tremors (resting, holding, intention), scanning dysarthria, cerebellar ataxia, nystagmus, bradykinesia, rigidity, gait disorder, and rarely, spastic epilepsy
Psychiatric manifestation [5]	Disorders of affects and impulses, depression, psychosis, behavior disorder
Kidneys [5]	Renal tubular acidosis, urolithiasis, proteinuria, tubular dysfunction
Eye [5]	Kayser-Fleischer corneal rings, sunflower cataract
Heart [5]	Arrhythmias, cardiomyopathy
Muscles/bones [5]	Rhabdomyolysis, osteoporosis, osteomalacia, degenerative diseases of spine
Skin [5]	Acanthosis nigricans, lunula

WD has been identified by the combination of three important biochemical parameters: decreased serum ceruloplasmin, decreased serum copper, and increased urinary copper excretion. The determination of exchangeable copper (CuEXC) offers an accurate and straightforward assessment of copper overload, providing important insights into the severity and spread of disease. For a conclusive diagnosis in children who are asymptomatic, the two mutations that cause the condition must be present. 

WD is distinguished from other forms of hepatic failure by the decreased levels of alkaline phosphatase, increased total bilirubin, and comparatively milder rise of liver enzymes (AST/ALT). Hemolysis due to free copper may also elevate total bilirubin. For chronic liver disease, the Model for End-Stage Liver Disease (MELD) and Child-Pugh scores assess severity. Non-invasive liver stiffness measurements and biochemical fibrosis scores require further validation in WD patients [12]. Brain MRI is critical for diagnosis and monitoring treatment. It shows hyperintense changes in the T2-weighted images in the basal ganglia, thalami, midbrain, and pons. The involvement is usually symmetrical. Brain atrophy is common and particularly involves the proximal brainstem and subcortical areas. Typical MRI findings are seen in almost all patients with neurological symptoms who are not initiated on drugs in contrast to only 20-30% patients who are asymptomatic. 

WD is managed by regulating copper intake and excretion to achieve normal copper homeostasis. Establishing a net negative copper balance by zinc supplementation, chelation therapy, and dietary changes is crucial. DPA is the most effective treatment for encouraging copper excretion. Since DPA interferes with pyridoxine metabolism, Vitamin B6 is co-administered with it [13]. Zinc induces metallothionein, which prevents the absorption of copper. It slows the disease progression initially, however, it was found to be inferior to chelators in delaying liver damage [13]. Although there is little evidence, a low-copper diet is recommended to minimize copper consumption. For the management of neurologic and psychiatric symptoms, a multidisciplinary approach involving speech, physical, and occupational therapy is advantageous. Long-term management of WD requires ongoing surveillance of liver function, copper levels, and compliance. Urinary copper excretion and ceruloplasmin levels are used as tools to observe the outcome of treatment and make necessary changes [10]. Studies indicate up to 45% of patients on current therapies struggle with long-term adherence, highlighting the need for monitoring compliance in all WD presentations. More recent methods of replacing mutant ATP7B genes through gene therapy have proven effective in animal models, and human applications are currently being investigated [14].

## Conclusions

Our case report delineates the diverse clinical presentations of WD among the two siblings and the familial dynamics of the condition. After being convinced that there was little to no danger of developing WD, the eldest sibling's condition improved significantly. The psychological impact of genetic risk assessment cannot be overlooked. We emphasize the need for holistic patient care encompassing medical and psychosocial support to improve patient outcomes in WD.
